# Dietary replacement of inorganic trace minerals with lower levels of organic trace minerals leads to enhanced antioxidant capacity, nutrient digestibility, and reduced fecal mineral excretion in growing-finishing pigs

**DOI:** 10.3389/fvets.2023.1142054

**Published:** 2023-05-25

**Authors:** Yunxia Xiong, Bailei Cui, Zhentao He, Shuai Liu, Qiwen Wu, Hongbo Yi, Fei Zhao, Zongyong Jiang, Shenglan Hu, Li Wang

**Affiliations:** ^1^State Key Laboratory of Livestock and Poultry Breeding, Key Laboratory of Animal Nutrition and Feed Science in South China Ministry of Agriculture, Maoming Branch, Guangdong Laboratory for Lingnan Modern Agriculture, Guangdong Key Laboratory of Animal Breeding and Nutrition, Institute of Animal Science, Guangdong Academy of Agricultural Sciences, Guangzhou, China; ^2^DeBon Bio-Tech Co., Ltd., Hengyang, Hunan, China

**Keywords:** organic trace minerals, growing-finishing pigs, antioxidant capacity, apparent nutrient digestibility, fecal mineral excretion

## Abstract

**Introduction:**

More effective and environment-friendly organic trace minerals have great potential to replace the inorganic elements in the diets of livestock. This study aimed to investigate the effects of dietary replacement of 100% inorganic trace minerals (ITMs) with 30–60% organic trace minerals (OTMs) on the performance, meat quality, antioxidant capacity, nutrient digestibility, and fecal mineral excretion and to assess whether low-dose OTMs could replace whole ITMs in growing-finishing pigs' diets.

**Methods:**

A total of 72 growing-finishing pigs (Duroc × Landrace × Yorkshire) with an initial average body weight of 74.25 ± 0.41 kg were selected and divided into four groups with six replicates per group and three pigs per replicate. The pigs were fed either a corn-soybean meal basal diet containing commercial levels of 100% ITMs or a basal diet with 30, 45, or 60% amino acid-chelated trace minerals instead of 100% ITMs, respectively. The trial ended when the pigs' weight reached ~110 kg.

**Results:**

The results showed that replacing 100% ITMs with 30–60% OTMs had no adverse effect on average daily gain, average daily feed intake, feed/gain, carcass traits, or meat quality (*P* > 0.05) but significantly increased serum transferrin and calcium contents (*P* < 0.05). Meanwhile, replacing 100% ITMs with OTMs tended to increase serum T-SOD activity (0.05 ≤ *P* < 0.1), and 30% OTMs significantly increased muscle Mn-SOD activity (*P* < 0.05). Moreover, replacing 100% ITMs with OTMs tended to increase the apparent digestibility of energy, dry matter, and crude protein (0.05 ≤ *P* < 0.1) while significantly reducing the contents of copper, zinc, and manganese in feces (*P* < 0.05).

**Discussion:**

In conclusion, dietary supplementation with 30–60% OTMs has the potential to replace 100% ITMs for improving antioxidant capacity and nutrient digestibility and for reducing fecal mineral excretion without compromising the performance of growing-finishing pigs.

## Introduction

Trace minerals are essential nutrients for animals to maintain normal health, growth, and reproduction (1). Owing to cost considerations, most mineral supplements currently used in livestock production are commonly derived from inorganic compounds, such as sulfates, oxides, chlorides, and carbonates. Researchers have found that fiber, phytate, tannin, oxalate, and silicate can interact with inorganic trace minerals (ITMs) in the gastrointestinal tract, resulting in low absorption and poor bioavailability of trace minerals. Due to the large safety margin of supplementation, mineral additives are often supplemented in animal feed in amounts greater than the physiological requirements to prevent deficiency and achieve maximum production potential (2). Hence, this excess supplementation can result in the excretion of trace minerals in feces, leading to soil and surface water pollution (3).

Another category of trace mineral additives is often referred to as organic trace minerals (OTMs). A growing body of evidence suggests that OTMs are more stable and less reactive in the digestive tract and can be better absorbed by the gut (3–5). Given the characteristics of high cost and high bioavailability, reducing the amount used while maintaining efficiency is an effective strategy for strengthening the promotion of OTMs in production. Dietary replacement of ITMs with lower levels of OTMs has been reported to have no negative impact on the performance of broilers (6), laying hens (7, 8), and weaned piglets (9) while reducing fecal mineral excretion, which is important for environmental protection. However, there has been little discussion on the effects of using lower levels of OTMs instead of ITMs on the performance of growing-finishing pigs, which consume the majority of the additive trace minerals in pig production.

Furthermore, most of the amino acid-chelated trace minerals used in the previous studies were single amino acid chelates, such as Gly-Fe, Gly-Zn, and Met-Zn (10–13). In this study, we used a novel compound, an amino acid-chelated trace mineral product, containing 18 types of amino acids. While OTMs have great potential to replace ITMs in animal feed, the extent to which this replacement is possible using the novel compound of amino acid-chelated trace minerals is not well defined.

A study using a similar OTM product (amino acid-chelated minerals) in laying hens has indicated that dietary supplementation with 30–50% amino acid-chelated trace minerals has the potential to replace 100% ITMs for improving eggshell quality, antioxidant capacity, and immune function (8). Therefore, we designed this study to evaluate the effects of dietary trace mineral sources (inorganic vs. amino acid-chelated) and concentrations (100% vs. 30–60%) on the performance, carcass traits, meat quality, antioxidant capacity, nutrient digestibility, and fecal mineral excretion in growing-finishing pigs. The study also aimed to assess whether low-dose OTMs could replace whole ITMs in the diets of growing-finishing pigs and to provide guidance for the application of OTMs in the livestock industry.

## Materials and methods

### Animal management, diets, and experimental design

A total of 72 growing-finishing pigs (Duroc × Landrace × Yorkshire) with an initial average body weight of 74.25 ± 0.41 kg were allocated to four treatments, as shown in Table 1, in a randomized complete block design based on body weight. All treatments were further subdivided into six replicates containing three pigs each. In addition to trace minerals, the basal diet (as shown in Table 2) was formulated to meet the standards set by the NRC (2012). For the experimental diets, the mineral premix was prepared using inorganic trace minerals of all components except Fe, Cu, Zn, and Mn. Supplementary ITMs in the control group diet (100% ITMs, sulfates) were formulated to be typical of those currently used in the Chinese pig industry, and the additional amounts of Fe, Cu, Zn, and Mn were 80, 15, 65, and 35 mg/kg, respectively. Organically complexed Fe, Cu, Zn, and Mn were separately added to the basal diet at proportions of 30, 45, and 60% (30% OTMs, 45% OTMs, and 60% OTMs) of those in the control group as MINEXO™ (Table 2). The trial ended when the pigs' weight reached ~110 kg. The feed consumption was recorded every week, and the body weight was monitored at the end of the experiment to calculate average daily gain (ADG), average daily feed intake (ADFI), and the ratio of feed to gain (F/G). The pigs were given *ad libitum* access to feed and water.

**Table 1:** Experiment design.

**Items**	**Forms**	**100% ITMs**	**OTMs**		
			**30%**	**45%**	**60%**
Fe, mg/kg	Amino acid-chelated	-	24	36	48
	Sulfates	80	-	-	-
Cu, mg/kg	Amino acid-chelated	-	4.5	7	9
	Sulfates	15	-	-	-
Zn, mg/kg	Amino acid-chelated	-	19.5	29	39
	Sulfates	65	-	-	-
Mn, mg/kg	Amino acid-chelated	-	10.5	16	21
	Sulfates	35	-	-	-

ITMs, inorganic trace minerals; OTMs, organic trace minerals.

**Table 2:** Composition and nutrient levels of experimental diets (air-dry basis).

**Ingredients**	**Content, %**	**Nutrient levels^b^**	**Content**
Corn	79.67	Digestive energy, kcal/kg	3050.03
Soybean meal (46%)	15.33	Crude protein, %	15.54
Soybean oil	1.39	Crude fiber, %	2.32
CaHPO_4_	0.90	Crude fat, %	2.25
Limestone	0.95	Neutral detergent fiber, %	8.62
NaCl	0.50	Acid detergent fiber, %	3.09
Choline chloride (50%)	0.10	Moisture, %	13.54
*L*-Lysine hydrochloride	0.21	Ash, %	5.14
*DL*-Methionine	0.02	Ca, %	0.59
*L-*Tryptophan	0.03	TP, %	0.22
Premix^a^	0.90	SID Lys, %	0.83
Total	100	SID Met + Cys, %	0.49
		SID Thr, %	0.50
		SID Trp, %	0.17

^a^The premix provided the following per kg of diets: VA 12 400IU, VD_3_ 2 800 IU, VE 30 IU, VK 5 mg, VB_12_ 40 μg, VB_1_ 3 mg, VB_2_ 10 mg, nicotinic acid 40 mg, D-pantothenic acid 15 mg, folic acid 1 mg, VB_6_ 8 mg, biotin 0.08 mg, CaI_2_O_6_ 0.92 mg, Na_2_SeO_3_ 0.77 mg.

^b^Nutrient levels were determined, except that digestible energy, SID Lys, SID Met, SID Thr, and SID Trp were calculated. The crude protein, crude fiber, crude fat, neutral detergent fiber, acid detergent fiber, moisture, ash, Ca, and TP were all determined according to the China National Standard, and the document numbers are GB/T 6432-2018, GB/T 6434-2006, GB/T 6433-2006, GB/T 20806-2006, NY/T 1459-2007, GB/T 6435-2014, GB/T 6438-2007, GB/T 6436-2002, and GB/T 6437-2018, respectively.

The amino acid-chelated Fe, Cu, Zn, and Mn were provided as MINEXO Fe™, MINEXO Cu™, MINEXO Zn™, and MINEXO Mn™ (DeBon Bio-tech Co., Ltd., Hunan, China), and amino acids and metal elements were chelated at 2:1 (moles). Moreover, the amino acids were produced from hydrolyzed plant protein and included 18 types of amino acids. MINEXO Fe™, MINEXO Cu™, MINEXO Zn™, and MINEXO Mn™ each contain 1.8 × 10^5^ mg/kg Fe, 1.8 × 10^5^ mg/kg Cu, 1.6 × 10^5^ mg/kg Zn, and 1.6 × 10^5^ mg/kg Mn, respectively. The measured values of natural Fe, Cu, Zn, and Mn in the basal diet were 105.21, 2.73, 22.97, and 57.55 mg/kg, respectively. The measured values of Fe, Cu, Zn, and Mn in all experimental diets are provided in Table 3.

**Table 3:** Measured values of trace minerals in experimental diets (air-dry basis).

**Items**	**100% ITMs**	**OTMs**		
		**30%**	**45%**	**60%**
Fe, mg/kg	173.00	136.50	154.00	163.00
Cu, mg/kg	17.30	10.50	12.10	13.90
Zn, mg/kg	91.60	42.90	54.50	62.60
Mn, mg/kg	92.10	66.70	77.30	83.60

ITMs, inorganic trace minerals; OTMs, organic trace minerals.

### Sample collection and measurements

Fresh feces were collected from each replicate for 3 consecutive days before the trial ended and stored at −20°C. After pooling, mixing, drying, and grinding, ~100 g of dried fecal samples were taken by the four-part method to determine the energy, dry matter, crude ash, crude protein, and titanium dioxide contents. At the end of the test, all pigs were weighed after a 12-h fasting period. Each replicate had one pig whose body weight was closest to the average body weight of the group slaughtered and sampled. Serum samples were harvested with non-anticoagulant tubes. Upon being electro-stunned, exsanguinated, dehaired, eviscerated, and split down the midline, the hot carcass, leaf fat, heart, liver, spleen, lung, and kidney were weighed, and the weight was recorded to calculate the dressing percentage and the organ index. The backfat thickness of the 1st rib, the 10th rib, the thoracolumbar junction, and the lumbar-sacral junction of the left carcass was measured with a vernier caliper, and the mean value was presented as the average backfat thickness. The loin eye muscle area was measured by tracing the transaction area at the thoracolumbar junction of the left carcass with transparent sulfate paper and calculated with a digital planimeter instrument (KP90N, Koizumi, Osaka, Japan). The muscle that spans from the fourth rib to the dorsal region near the hip was removed from the left carcass for meat quality analysis. Tissue samples of liver and muscle from the same part were collected and frozen at −80°C.

### Meat quality determination

The method of analysis of meat quality was based on previous studies with some modifications (14–16). Briefly, meat color was measured at 45 min, 24 h, and 48 h postmortem using a D-65 light source portable chroma meter (CR-410, Minolte, Chiyoda, Japan). Moreover, pH was determined at 45 min, 24 h, and 48 h postmortem using a portable pH meter (testo-205, Testo, Lenzkirch, Germany). Drip loss was evaluated by suspending the muscle samples with a fishhook in an aerated polyethylene film bag. Marbling scores were scored by 10 assessors against the standard scoring chart for pork marble (National Pork Producers Council, 1991). Shear force was measured with an Instron machine (Instron model 4411, Instron Corp., Canton, MA) equipped with a Warner-Bratzler blade.

### Serum biochemical parameter measurement

Serum biochemical parameters including serum glucose (GLU, cat: 200891), total protein (TP, cat: 180531), albumin (ALB, cat: 180641), urea (URE, cat: 100000280), triglyceride (TG, cat: 198021), total cholesterol (CHO, cat: 192061), high-density lipoprotein (HDL-C, cat: 100020235), low-density lipoprotein (LDL-C, cat: 100020245), alanine aminotransferase (ALT, cat: 100020000), aspartate aminotransferase (AST, cat: 100020010), total bilirubin (TBIL, cat: 201171), alkaline phosphatase (AKP, cat: 100020020), creatinine (CRE, cat: 100020170) were measured using commercial kits purchased from Biosino Bio-technology & Science, Inc. (Beijing, China) by using an automatic biochemical analyzer (Selectra Pro XL, EliTechGroup, Puteaux, France). Porcine transferrin (TRF, cat: H130) and ceruloplasmin (CP, cat: A029-1-1) were detected using commercial kits purchased from Nanjing Jiancheng Bioengineering Institute (Nanjing, China).

### Antioxidant capacity evaluation

Malondialdehyde (MDA, cat: A003-2), total antioxidant capacity (T-AOC, cat: A015-2-1), total superoxide dismutase (T-SOD, cat: A001-2), copper-zinc superoxide dismutase (Cu/Zn-SOD, cat: A001-2), manganese superoxide dismutase (Mn-SOD, cat: A001-2), catalase (CAT, cat: A007-1-1), and glutathione peroxidase (GSH-px cat: A005) were evaluated using commercial kits purchased from Nanjing Jiancheng Bioengineering Institute (Nanjing, China). Samples of liver and muscle tissues were homogenized with physiological saline, and the supernatant was harvested after centrifugation for later detection. The final data were normalized to total tissue protein concentration.

### Apparent nutrient digestibility detection

The quantity of dry matter, crude ash, crude protein, and titanium dioxide in fecal samples and diets were determined according to the China National Standard, and the document numbers are GB/T 6435-2014, GB/T 6438-2007, GB/T 6432-2018, and GB5009.246-2016, respectively. The apparent digestibility of energy, dry matter, crude ash, and crude protein was calculated using the following formula:


(1) $$\begin{array}{l} \text{Apparent nutrient digestibility\%}=\left(1-\frac{\text{Nutrient content in the feces}\times \text{Titanium dioxide content in the diet}}{\text{Nutrient content in the diet}\times \text{ Titanium dioxide content in the feces}}\right)\times 100\text{\% } \end{array}$$

### Fecal excretion analysis

The quantity of crude ash, N, P, and Ca in fecal samples was determined according to the China National Standard, and the document numbers are GB/T 6438-2007, GB/T 6432-2018, GB/T 6436-2002, and GB/T 6437-2018, respectively. However, Fe, Cu, Zn, and Mn were measured according to GB 5009.268-2016.

### Statistical analysis

Data management and analysis were performed using the IBM SPSS Statistics V18.0 software package (IBM Corp., Armonk, NY, USA), and the results were presented as the mean and pooled standard error. Before performing the difference analysis between groups, the Shapiro–Wilk test was performed to verify the normality of the data. When the variable data presented a non-normal distribution, a one-way ANOVA, followed by the Kruskal–Wallis test, was used for analysis with multiple FDR corrections. When the data presented a normal distribution, a one-way ANOVA analysis followed by an LSD test was used. Differences were considered significantly expressed at a *P*-value of < 0.05 and with a significant tendency at 0.05 ≤ *P* < 0.10.

## Results

### Performance, carcass traits, and organ index

As shown in Tables 4, 5, no differences in growth performance, carcass traits, or organ index parameters were observed after growing-finishing pigs were treated with lower levels of organic Fe, Cu, Zn, and Mn compared to inorganic forms (*P* > 0.05).

**Table 4:** Effects of the dietary replacement of inorganic trace minerals with lower levels of organic trace minerals on the growth performance and carcass traits of growing-finishing pigs.

**Items**	**100% ITMs**	**OTMs**			**SEM**	***P*-value**
		**30%**	**45%**	**60%**		
Initial BW, kg	74.28	74.25	74.22	74.25	0.117	0.999
Final BW, kg	109.54	110.81	110.21	109.96	0.502	0.860
ADG, kg/d	0.97	0.96	0.96	0.97	0.012	0.991
ADFI, kg/d	3.15	3.16	3.17	3.16	0.025	0.998
F/G	3.27	3.29	3.30	3.25	0.038	0.980
Carcass weight, kg	82.62	81.74	80.60	81.32	0.658	0.766
Dressing percentage, %	75.34	75.17	75.65	74.94	0.550	0.979
Loin eye muscle area, cm^2^	57.28	62.61	63.62	58.10	1.678	0.463
Average back-fat thickness, mm	23.54	21.50	23.40	22.36	0.658	0.701
Leaf fat weight, kg	1.07	0.99	1.06	1.07	0.497	0.914

Values are presented as mean and pooled SEM, *n* = 6.

Data in the same row with no or the same letter indicate no significant difference (*P* > 0.05), while with different letters mean significant difference (*P* < 0.05).

ITMs, inorganic trace minerals; OTMs, organic trace minerals; BW, body weight; ADG, average daily gain; ADFI, average daily feed intake; F/G, feed-to-gain ratio.

**Table 5:** Effects of dietary replacing inorganic with lower levels of organic trace minerals on the organ index of finishing pigs (%).

**Items**	**100% ITMs**	**OTMs**			**SEM**	***P*-value**
		**30%**	**45%**	**60%**		
Heart index	0.37	0.37	0.37	0.40	0.009	0.765
Liver index	1.49	1.54	1.53	1.61	0.031	0.601
Spleen index	0.21	0.21	0.21	0.23	0.011	0.882
Lung index	0.82	1.05	0.85	0.90	0.043	0.247
Kidney index	0.29	0.32	0.28	0.32	0.008	0.186

Values are presented as mean and pooled SEM, *n* = 6.

Data in the same row with no or the same letter indicate no significant difference (*P* > 0.05), while with different letters mean significant difference (*P* < 0.05).

ITMs, inorganic trace minerals; OTMs, organic trace minerals.

### Meat quality

As shown in Table 6, compared with the 100% ITM group, ~30–60% OTMs had no significant effect on meat color, pH, drip loss, shear force, marble score, or moisture content (*P* > 0.05).

**Table 6:** Effects of the dietary replacement of inorganic trace minerals with lower levels of organic trace minerals on the meat quality traits of growing-finishing pigs.

**Items**	**100% ITMs**	**OTMs**			**SEM**	***P*-value**
		**30%**	**45%**	**60%**		
**Meat color**						
L${}_{45\text{min}}^{*}$	43.75	45.39	44.26	44.81	0.313	0.290
a${}_{45\text{min}}^{*}$	16.66	15.72	15.84	16.35	0.225	0.428
b${}_{45\text{min}}^{*}$	6.17	5.97	5.91	6.25	0.109	0.666
L${}_{24\text{h}}^{*}$	57.02	57.53	57.19	56.43	0.349	0.750
a${}_{24\text{h}}^{*}$	15.83	15.54	15.46	15.62	0.169	0.894
b${}_{24\text{h}}^{*}$	4.83	4.60	5.37	5.53	0.452	0.884
L${}_{48\text{h}}^{*}$	57.15	57.88	57.87	56.93	0.356	0.724
a${}_{48\text{h}}^{*}$	16.20	15.95	16.16	16.46	0.168	0.787
b${}_{48\text{h}}^{*}$	3.05	2.50	2.68	3.05	0.118	0.263
**pH value**						
pH_45min_	6.36	6.34	6.26	6.34	0.025	0.562
pH_24h_	5.44	5.46	5.43	5.47	0.011	0.508
pH_48h_	5.46	5.46	5.44	5.48	0.010	0.494
**Drip loss**						
Drip loss_24h_	3.12	2.92	3.11	2.93	0.077	0.713
Drip loss_48h_	3.75	3.86	3.93	3.84	0.123	0.970
Shear force, *N*	52.15	57.96	59.45	59.88	2.082	0.555
Marbling score	2.659	2.603	2.619	2.691	0.131	0.996
Moisture, %	73.73	74.35	74.39	74.09	0.116	0.403

Values are presented as mean and pooled SEM, *n* = 6.

Data in the same row with no or the same letter indicate no significant difference (*P* > 0.05), while with different letters mean a significant difference (*P* < 0.05).

ITMs, inorganic trace minerals; OTMs, organic trace minerals; L^*^, lightness; a^*^, redness; b^*^, yellowness.

### Serum biochemical parameters

Table 7 shows that replacing 100% ITMs with dietary supplementation with reduced OTMs had no significant effect on the contents of serum glucose, total protein, albumin, urea, cholesterol, low-density lipoprotein, high-density lipoprotein, alanine aminotransferase, aspartate transferase, total bilirubin, alkaline phosphatase, creatinine, ceruloplasmin, and phosphorus (*P* > 0.05). Serum triglyceride, transferrin, and calcium contents were significantly higher in the OTM groups compared to the 100% ITM group (*P* < 0.05).

**Table 7:** Effects of the dietary replacement of inorganic trace minerals with lower levels of organic trace minerals on the serum biochemical parameters of growing-finishing pigs.

**Items**	**100% ITMs**	**OTMs**			**SEM**	***P*-value**
		**30%**	**45%**	**60%**		
GLU, mM/L	4.67	4.62	5.09	4.53	0.154	0.616
TP, g/L	65.95	65.93	67.37	69.86	0.955	0.377
ALB, g/L	22.07	24.12	26.25	27.49	0.845	0.105
URE, mM/L	8.32	7.75	10.09	9.47	0.390	0.137
TG, mM/L	0.33^b^	1.70^a^	1.71^a^	1.69^a^	0.173	0.002
CHO, mM/L	2.15	2.04	2.04	1.97	0.052	0.691
HDL-C, mM/L	0.77	0.88	0.95	0.94	0.034	0.241
LDL-C, mM/L	0.94	0.71	0.88	0.88	0.033	0.106
ALT, IU/L	42.03	41.27	47.63	39.64	1.337	0.167
AST, IU/L	50.54	41.45	43.67	44.41	1.686	0.270
TBIL, μM/L	4.66	9.11	12.21	9.18	1.001	0.064
AKP, IU/L	117.24	125.16	125.48	122.64	3.927	0.885
CRE, μM/L	143.22	159.70	160.01	158.20	3.148	0.175
TRF, mg/mL	2.05^b^	2.75^a^	2.94^a^	3.03^a^	0.128	0.017
CP, IU/L	61.07	60.90	65.72	67.20	1.910	0.578
Ca, mM/L	1.41^b^	1.54^a^	1.61^a^	1.61^a^	0.024	0.005
P, mM/L	0.44	0.46	0.45	0.42	0.010	0.611

Values are presented as mean and pooled SEM, *n* = 6.

Data in the same row with no or the same letter indicate no significant difference (*P* > 0.05), while data with different letters indicate a significant difference (*P* < 0.05).

ITMs, inorganic trace minerals; OTMs, organic trace minerals; GLU, glucose; TP, total protein; ALB, albumin; URE, urea; TG, triglyceride; CHO, cholesterol; HDL-C, high-density lipoprotein cholesterol; LDL-C, low-density lipoprotein cholesterol; ALT, alanine aminotransferase; AST, aspartate transaminase; TBIL, total bilirubin; AKP, alkaline phosphatase; CRE, creatinine; TRF, transferrin; CP, ceruloplasmin.

### Antioxidant capacity

As shown in Table 8, the OTM groups tended to increase serum T-SOD activity compared with the 100% ITMs group (0.05 ≤ *P* < 0.1), and 30% OTMs significantly increased muscle Mn-SOD activity (*P* < 0.05). However, there was no difference in hepatic antioxidant capacity between the ITM and OTM groups (*P* > 0.05).

**Table 8:** Effects of the dietary replacement of inorganic trace minerals with lower levels of organic trace minerals on the antioxidant capacity of growing-finishing pigs.

**Items**	**100% ITMs**	**OTMs**			**SEM**	***P*-value**
		**30%**	**45%**	**60%**		
**Serum**						
MDA, nM/mL	2.12	1.88	1.81	2.01	0.208	0.957
T-AOC, mM/L	0.33	0.34	0.33	0.35	0.003	0.269
T-SOD, IU/mL	193.33	218.08	216.23	221.91	4.337	0.063
Cu/Zn-SOD, IU/mL	146.54	157.68	165.89	170.16	4.197	0.218
Mn-SOD, IU/mL	46.78	60.40	51.46	55.24	4.182	0.703
CAT, IU/mL	3.06	2.87	2.96	3.17	0.227	0.972
GSH-px, IU/L	422.71	444.76	450.92	457.43	6.464	0.226
**Liver**						
MDA, nM/mg prot	0.39	0.50	0.49	0.50	0.021	0.145
T-AOC, mM/g prot	0.07	0.08	0.07	0.08	0.003	0.699
T-SOD, IU/mg prot	697.79	753.97	753.63	754.64	15.624	0.489
Cu/Zn-SOD, IU/mg prot	596.84	625.14	622.92	627.99	123.120	0.833
Mn-SOD, IU/mg prot	100.96	128.83	130.71	126.65	103.632	0.578
CAT, IU/mg prot	49.42	53.81	62.61	57.83	3.365	0.590
GSH-px, IU/mg prot	31.74	36.64	37.01	39.01	1.094	0.112
**Muscle**						
MDA, nM/mg prot	0.09	0.12	0.09	0.08	0.006	0.152
T-AOC, mM/g prot	0.04	0.04	0.04	0.04	0.006	0.534
T-SOD, IU/mg prot	120.58	122.97	123.30	122.76	2.267	0.976
Cu/Zn-SOD, IU/mg prot	88.93	80.01	85.67	84.50	2.322	0.592
Mn-SOD, IU/mg prot	31.65^b^	42.96^a^	37.62^ab^	38.26^ab^	1.497	0.039
CAT, IU/mg prot	0.31	0.37	0.57	0.46	0.052	0.331
GSH-px, IU/mg prot	2.82	3.63	4.42	4.60	0.492	0.584

Values are presented as mean and pooled SEM, *n* = 6.

Data in the same row with no or the same letter indicate no significant difference (*P* > 0.05), while data with different letters indicate a significant difference (*P* < 0.05).

ITMs, inorganic trace minerals; OTMs, organic trace minerals; MDA, malondialdehyde; T-AOC, total antioxidant capacity; T-SOD, total superoxide dismutase; CuZn-SOD, copper-zinc superoxide dismutase; Mn-SOD, manganese superoxide dismutase; CAT, catalase; GSH-px, glutathione peroxidase; prot, protein.

### Apparent nutrient digestibility

The data presented in Table 9 indicated that replacing 100% ITMs with OTMs tended to improve the apparent digestibility of energy, dry matter, and crude protein (0.05 ≤ *P* < 0.1).

**Table 9:** Effects of the dietary replacement of inorganic trace minerals with lower levels of organic trace minerals on the nutrient digestibility of growing-finishing pigs.

**Items**	**100% ITMs**	**OTMs**			**SEM**	***P*-value**
		**30%**	**45%**	**60%**		
Energy, %	85.69	85.51	87.96	87.85	0.364	0.065
Dry mass, %	83.60	84.18	86.05	85.77	0.403	0.075
Crude protein, %	76.75	78.40	81.17	79.83	0.656	0.088
Crude ash, %	22.58	22.81	29.86	33.92	2.117	0.152

Values are presented as mean and pooled SEM, *n* = 6.

Data in the same row with no or the same letter indicate no significant difference (*P* > 0.05), while data with different letters indicate a significant difference (*P* < 0.05).

ITMs, inorganic trace minerals; OTMs, organic trace minerals.

### Fecal excretion

The results of fecal excretion are shown in Table 10. Replacement of 100% ITMs with OTMs significantly reduced the fecal emissions of Cu, Zn, and Mn (*P* < 0.05). Moreover, the contents of crude ash and P were significantly lower in the 45 and 60% OTM groups than those in the 100% ITM group (*P* < 0.05).

**Table 10:** Effects of the dietary replacement of inorganic trace minerals with lower levels of organic trace minerals on the fecal excretion of growing-finishing pigs.

**Items**	**100% ITMs**	**OTMs**			**SEM**	***P*-value**
		**30%**	**45%**	**60%**		
Crude ash, %	24.28^a^	23.54^ab^	22.43^b^	20.50^c^	0.424	0.001
N, %	3.15	2.95	2.92	3.02	0.048	0.365
Ca, %	3.18	3.00	3.43	2.93	0.082	0.139
P, %	1.92^a^	1.84^ab^	1.69^bc^	1.59^c^	0.044	0.025
Fe, mg/kg	1836.00	1430.00	1469.20	1357.50	107.002	0.388
Cu, mg/kg	159.00^a^	92.73^c^	101.67^bc^	114.50^b^	5.745	< 0.001
Zn, mg/kg	846.67^a^	395.33^c^	418.00^c^	521.00^b^	38.754	< 0.001
Mn, mg/kg	762.67^a^	609.17^b^	635.50^b^	671.33^b^	18.169	0.007

Values are presented as mean and pooled SEM, *n* = 6.

Data in the same row with no or the same letter indicate no significant difference (*P* > 0.05), while data with different letters indicate a significant difference (*P* < 0.05).

ITMs, inorganic trace minerals; OTMs, organic trace minerals.

## Discussion

In the pig industry, excessive amounts of trace minerals are frequently added to pig diets to prevent deficiency. Apart from the supplemental amount, the natural trace minerals present in the raw feed materials are not low (17–20). According to the NRC (2012), the trace mineral requirements of the pigs in this study (70–110 kg) were Fe 41.9, Cu 3.09, Zn 50, and Mn 2 mg/kg, respectively, and the natural contents of trace minerals in the basal diet were Fe 105.21, Cu 2.73, Zn 22.97, and Mn 57.55 mg/kg, among which the contents of Fe and Mn had already fully met the requirements of experimental pigs. Moreover, all experimental diets in this study met or exceeded trace mineral requirements based on the NRC (2012) after inorganic or organic trace mineral supplementation, except for Zn content in the 30% OTM group's diet (42.9 mg/kg). However, since the NRC recommendations are based on inorganic trace minerals, we believe that they may not be fully applicable to organic trace minerals. In addition, supplementation with ITMs (Fe, Cu, Zn, and Mn) at 50% of the NRC (2012) recommendations had no negative effect on the growth performance, carcass traits, or the meat quality of the pigs (21). Therefore, we believed that the trace minerals in this study's experimental diets were in accordance with the animals' requirements.

As a more bioavailable source of trace minerals (22), OTMs are widely used in the production of pigs (12, 23), broilers (24, 25), laying hens (7, 8, 26), and dairy cows (27, 28) for their performance-enhancing effects. However, studies on animals fed different trace mineral sources have yielded inconsistent results. According to some studies, a significant reduction in ITM supplementation had no negative effect on the performance or meat quality parameters (6, 7, 9, 26, 29). For example, replacing ITMs with lower levels of OTMs had no significant effect on weight gain, feed intake, or carcass traits of pigs from weaning to slaughter (30), and the results of Zhang et al. (9) also indicated that replacing 100% ITMs with one-third of OTMs did not adversely affect the growth performance of piglets. Furthermore, replacing 100% ITMs with 50% OTMs reduced F/G and fecal mineral emissions without decreasing weight gain or feed intake in broilers (31), while 50% protein-chelated trace minerals instead of 100% ITMs decreased F/G in nursery piglets (23). Consistent with previous studies, we also found that adding 30% OTMs instead of 100% ITMs did not appear to have any adverse effect on the growth performance or carcass traits of growing-finishing pigs. The amino acid-chelated OTM product used in this study contains 18 types of amino acids, which are hydrolysates of natural plant protein with good absorption even at low doses. However, the results of this trial also demonstrated that excessive supplementation had no further beneficial effect on performance when the supplementation of OTMs was already fully meeting the pigs' dietary requirements.

Dietary supplementation with OTMs has the potential to improve meat quality characteristics. It has been reported that amino acid-chelated zinc supplementation could enhance cell proliferation and differentiation in myoblasts (10). Moreover, OTMs could reduce drip loss by 4–6 days postmortem, which is important for prolonging the shelf life of pork (29). The reduction or equivalent replacement of ITMs with OTMs has been reported to increase the L^*^ value and decrease the a^*^ value of chicken breast meat (32). In this study, the OTMs were found to have no adverse effect on pH, meat color, or drip loss, even when reduced to 30% of ITMs. The discrepancy between the results of the previous study and our study may be because the drip loss measurement in this experiment was only carried out 48 h postmortem, and the observation time for meat quality indicators may be insufficient.

Fe, Cu, Zn, and Mn are important components of some antioxidant enzymes. For example, Cu, Zn, and Mn are key cofactors of SOD. Studies have shown that OTMs have a higher digestibility and absorption rate compared with ITMs, which could improve SOD activity and reduce lipid oxidation (12, 13, 29, 33). Cows fed diets containing OTMs had higher serum SOD and GSH-px activities than those fed diets containing the same amount of ITMs (28), and pigs fed diets supplemented with OTMs had higher hepatic Cu/Zn-SOD and GSH-px activities than those fed diets supplemented with ITMs (34). Feng et al. (12) found that feeding diets supplemented with Fe-Gly could increase serum SOD activity in pigs. Does replacing inorganic trace minerals with lower levels of organic minerals weaken the antioxidant defense system? Zhang et al. (9) found that replacing 100% ITMs with 1/3 OTMs had no negative effect on serum T-AOC and MDA contents, as well as CAT, T-SOD, and Cu/Zn-SOD activities. Moreover, the results of this study indicated that the OTMs could enhance the antioxidant capacity by increasing the serum T-SOD activity and muscle Mn-SOD activity in growing-finishing pigs, even when the supplementation level was reduced to 30%.

Because they share the same absorption pathways as amino acids, amino acid-chelated minerals have a higher absorption rate than ITMs, less competition for ITM binding sites, and less excretion through the bile and feces (7, 23, 35). Supplementation with OTMs could improve the digestibility of Zn, Fe, and P in a corn-soybean meal diet for pigs (36). In agreement with the previous study, we also found that replacing ITMs with lower levels of OTMs resulted in higher apparent digestibility of energy, dry matter, and crude protein, lower emissions of fecal minerals, crude ash, and phosphorus, all of which are important for environmental protection.

## Conclusions

Therefore, our results demonstrate that dietary supplementation with 30–60% amino acid-chelated trace minerals has the potential to replace 100% ITMs to improve the antioxidant capacity and apparent nutrient digestibility and reduce fecal mineral excretion without affecting the performance of growing-finishing pigs.

## Data availability statement

The original contributions presented in the study are included in the article/supplementary material, further inquiries can be directed to the corresponding authors.

## Ethics statement

The animal study was reviewed and approved by Animal Care and Use Committee of Guangdong Academy of Agricultural Sciences (GAASIAS-2022-022). Written informed consent was obtained from the owners for the participation of their animals in this study.

## Author contributions

LW, FZ, and ZJ conceived and designed the trial. BC and ZH executed animal breeding and management. YX, SL, HY, and QW performed the sampling and parameter measurements. YX and SH analyzed the data and wrote the manuscript. All authors approved the submitted version.
